# Supplementary material for: Species identification and phylogenetic analysis of Leishmania isolated from patients, vectors and hares in the Xinjiang Autonomous Region, The People’s Republic of China
Source: PLoS Negl Trop Dis. 2021 Dec 17;15(12):e0010055. doi: 10.1371/journal.pntd.0010055 (PMC8752017; doi:10.1371/journal.pntd.0010055)
Supplement: Attachment — Submitted filename: reply to reviewers GH.docx [file pntd.0010055.s001.docx]

Dear Mr Lai,

Thank you very much for submitting your manuscript "Species identification and phylogenetic analysis of Leishmania isolated from patients, vectors and hares in the Xinjiang Autonomous Region, The People's Republic of China" for consideration at PLOS Neglected Tropical Diseases. As with all papers reviewed by the journal, your manuscript was reviewed by members of the editorial board and by several independent reviewers. In light of the reviews (below this email), we would like to invite the resubmission of a significantly-revised version that takes into account the reviewers' comments.
 
All reviewers came to the conclusion that this is an very interessant study, which has however some restrictions e.g. small sample size and limited number of loci studied. I think the authors should try to address these comments in the revised manuscript. For instance comparison of their data to data from Indian L. donovani as suggested by reviewer 2 seems to be feasible. I also agree with reviewer 1 that it would be very interesting to perform whole-genome sequencing of these Chinese strains and to compare these data with the numerous L. donovani/ L. infantum sequences already available . But this could be perhaps w be published in a next manuscript. The present study could be considered as a pilot study. The authors need however to discuss the restrictions and future prospects of their work in this submission.


We cannot make any decision about publication until we have seen the revised manuscript and your response to the reviewers' comments. Your revised manuscript is also likely to be sent to reviewers for further evaluation.
 
When you are ready to resubmit, please upload the following:

[1] A letter containing a detailed list of your responses to the review comments and a description of the changes you have made in the manuscript. Please note while forming your response, if your article is accepted, you may have the opportunity to make the peer review history publicly available. The record will include editor decision letters (with reviews) and your responses to reviewer comments. If eligible, we will contact you to opt in or out.

[2] Two versions of the revised manuscript: one with either highlights or tracked changes denoting where the text has been changed; the other a clean version (uploaded as the manuscript file).

Important additional instructions are given below your reviewer comments.

Please prepare and submit your revised manuscript within 60 days. If you anticipate any delay, please let us know the expected resubmission date by replying to this email. Please note that revised manuscripts received after the 60-day due date may require evaluation and peer review similar to newly submitted manuscripts.
 
Thank you again for your submission. We hope that our editorial process has been constructive so far, and we welcome your feedback at any time. Please don't hesitate to contact us if you have any questions or comments.
 
Sincerely,

Gabriele Schönian
Associate Editor
PLOS Neglected Tropical Diseases

Michael Boshart
Deputy Editor
PLOS Neglected Tropical Diseases

***********************

All reviewers came to the conclusion that this is an very interessant study, which has however some restrictions e.g. small sample size and limited number of loci studied. I think the authors should try to address these comments in the revised manuscript. For instance comparison of their data to data from Indian L. donovani as suggested by reviewer 2 seems to be feasible. I also agree with reviewer 1 that it would be very interesting to perform whole-genome sequencing of these Chinese strains and to compare these data with the numerous L. donovani/ L. infantum sequences already available . But this could be perhaps w be published in a next manuscript. The present study could be considered as a pilot study. The authors need however to discuss the restrictions and future prospects of their work in this submission.

>>Thanks for the fair comments and suggestions. We have addressed these two major concerns point-by-point replies.

Reviewer's Responses to Questions

<b>Key Review Criteria Required for Acceptance?</b></br></br>
As you describe the new analyses required for acceptance, please consider the following:</br></br>

<b>Methods</b></br></br>
-Are the objectives of the study clearly articulated with a clear testable hypothesis stated?</br>
-Is the study design appropriate to address the stated objectives?</br>
-Is the population clearly described and appropriate for the hypothesis being tested?</br>
-Is the sample size sufficient to ensure adequate power to address the hypothesis being tested?</br>
-Were correct statistical analysis used to support conclusions?</br>
-Are there concerns about ethical or regulatory requirements being met?</br></br>

Reviewer #1: With cultures available, it may be relatively easy to sequence genomes of all these 20 isolates. The downstream analysis would make all the conclusions stronger. The presented analyses (based on 3 loci, some of which were not successfully amplified across the dataset - f.e. nagt) should be considered preliminary.

Reviewer #1: The presented results should be further complemented (or replaced altogether) by the analyses based on whole genome sequences.

Reviewer #1: See above. I believe the phylogenomic analyses will make the manuscript much stronger.

>> Thanks a lot for the comments and suggestions. Concerning the genomic studies, we totally agree that it would be very interesting to perform. However, it would need to be published in a next manuscript as currently we have limited funding to carry out this project. The present study could be considered as a pilot study, and we have discussed the restrictions and future prospects for this work.

Reviewer #2: Objective is clear as stated
The sample size is a bit small
Sequence phylogenetic analysis based on MEGA version 5
Met ethical/regulatory requirements

>> Thanks for the positive comments.

--------------------

<b>Results</b></br></br>
-Does the analysis presented match the analysis plan?</br>
-Are the results clearly and completely presented?</br>
-Are the figures (Tables, Images) of sufficient quality for clarity?</br></br>

Reviewer #2: Phylogenetic analyses are standard
Figures and Tables are acceptable

>> Thanks for the positive comments.

--------------------

<b>Conclusions</b></br></br>
-Are the conclusions supported by the data presented?</br>
-Are the limitations of analysis clearly described?</br>
-Do the authors discuss how these data can be helpful to advance our understanding of the topic under study?</br>
-Is public health relevance addressed?</br></br>


Reviewer #2: Conclusions acceptable, but weak due to small sample size
Discussion includes statements for the limitations of the analyses
Significance of the data obtained was discussed
Public health is not part of the work, but relevant points mentioned

>> Thanks for the positive comments. The sample size was largely due to the status of leishmaniasis, which is currently comprehensively under control in China. Both patient and animal infections are rare, therefore, isolates obtained are very limited, these are all isolates that we collected over 10 years. We have added information in the limitations of the analyses and we hope that the reviewer can kindly appreciate this situation.

--------------------

<b>Editorial and Data Presentation Modifications?</b></br><br/>
Use  this section for editorial suggestions as well as relatively minor modifications of existing data that would enhance clarity. If the only modifications needed are minor and/or editorial, you may wish to recommend “Minor Revision” or “Accept”.

Reviewer #2: P. 3, last para., 1st sentence: Clarify the description of DT-ZVL, which has been categorized into sandy desert type and pebble desert type with different sand fly vectors in Xinjiang.

>> Thanks for the comment, we have rewritten the sentence to clarify DT-ZVL in the text.

P. 4, lines 5-6: Define what are "Leishmania antigens" and "typical VL symptoms" besides splenomegaly ?

>> “Leishmania antigens” means rK39, it is a recombinant product of the 39 amino acid repeats found in a kinesin-like gene of visceral *Leishmania* spp. Also see reference: Qu JQ, Zhong L, Masoom-Yasinzai M, Abdur-Rab M, Aksu HSZ, Reed SG, Chang K-P, Gilman-Sachs A. Serodiagnosis of Asian leishmaniasis with a recombinant antigen from the repetitive domain of a Leishmania kinesin［J］.Trans R Soc Trop Med Hyg，1994， 88 （5）：543-545.

"typical VL symptoms" of infected hare includes splenomegaly and hepatomegaly, moreover, some hares also have infection in the ear, leading to ulcerative lesions. We have added this into the text.

P. 5, last 3 lines: "unknown reasons" and poor "sample quality" are inconsistent statements

>> Thanks for the comment, we have revised it in the text.

P. 6, 3rd para., line 1: "A/G substitution" is inconsistent with nts shown in position 78 in Fig. 3 ?

>> Sorry for the mistake, we have corrected it in the text.

P. 6, last para., 1st line: Is hsp70 encoded by tandem-repeated genes in Leishmania ? If so, the negative PCR is surprising for 4 of the 20 samples examined.

>> The hsp70 genes are tandemly repeated gene, the failure of the amplification in the 4 sample might be due to the poor quality of these samples preserved (in 70% ethanol for too long), we had tried alternative primer sets but still failed to amplify the sequence.

P. 8, 2nd para., line 5-8: Several points here need attention for clarification. L. infantum has long been considered as the causative species of AVL in the Kashi alluvial plain based on DNA/sequence analyses. It is incorrect to say "no animal reservoir has been found for the Chinese L. infantum". Dog has been clearly shown as a reservoir and racoon dog considered as another one for infantum VL in eastern China. It may be acceptable to replace "Chinese" with "Xinjiang" in that statement.

P. 8, last sentence: The same problem as stated above. Negative finding in search for animal reservoirs also cannot be conclusive.

P. 9, 1st para. line 10 and 2nd para., line 4: Replace "China" and "Chinese" with Xinjiang in these two statement to avoid the same problem.

>> Many thanks for the above comments, we have revised the accordingly.

P. 10,Line 8: "bug" "Largus largus" ? Do you mean Lagurus lagurus ? If so, it is not a bug !?
>> Sorry for the mistake and carelessness, we have deleted the “bug” and Largus largus was changed to *Lagurus lagurus* in the text.

--------------------

<b>Summary and General Comments</b></br></br>
Use  this section to provide overall comments, discuss strengths/weaknesses of the study, novelty, significance, general execution and scholarship. You may also include additional comments for the author, including concerns about dual publication, research ethics, or publication ethics. If requesting major revision, please articulate the new experiments that are needed.

Reviewer #2: The work presented is highly significant by studying Leishmania rarely examined in an endemic area of VL with unique epidemiology. The authors correctly pointed out in the Discussion that amastigotes should be examined for genomic phylogenetic analyses. Such data from amastigotes of Indian L donovani clinical samples have been published. Their hsp70, ITS1 and nagt sequences should be included for phylogenetic analysis in this study. The relative merits of the three sequence markers used should be discussed with reference to their copy number per haploid genome. It is further suggested to include a brief discussion for future experimental study to verify the role of Tarim hare as a reservoir of DT-ZVL.

>>Many thanks for the comments. We referred to the literature and obtained NAGT sequence from MHOM/IN/1983/AG83, ITS1 sequence from MHOM/IN/00/DEVI, and Hsp70 sequence from MHOM/IN/80/DD8. However, these sequences were found to be 100% identical to the ones already present in the corresponding phylogenetic analysis (the NAGT sequence from HOM/IN/97/JD, the ITS1 sequence from MHOM/IN/80/DD8, and the Hsp70 sequence from MHOM/IN/00/DEVI). Therefore, we think that it is unnecessary to include them as India representative sequences. However, we have included this idea in the limitations section and explained the similarity.

We have also added a few sections in the limitations section (page 8) to cover the comments on the merits of the three sequence markers used and some brief discussion of the future studies needed to verify the role of the Tarim Hare.

Reviewer #3: The manuscript “Species identification and phylogenetic analysis of Leishmania isolated from patients, vectors and hares in the Xinjiang Autonomous Region, The People's Republic of China” applies a molecular epidemiological approach on 20 new field isolates obtained from two endemic sites, which are analysed for the three diagnostic genes ITS1, HSP70 and NAGT by PCR amplification, sequencing and phylogenetic mapping. The manuscript provides important new epidemiological information by (i) distinguishing both L. infantum and L. donovani infection in the Xinjiang Autonomous Region, (ii) demonstrating a non-classical, anthroponotic transmission cycle for L. infantum and a non-classical, zoonotic transmission cycle for L. donovani isolates, and (iii) involving hares as novel reservoir for L. donovani. Overall, the paper is very well written, and the drawn conclusions are fully supported by the results. Thus, I do not have any major criticism. One minor comment is that often the authors write that leishmaniasis is transmitted by sand flies, yet it is the parasite and not the disease that is transmitted. Also, the authors may consider combining the haplotype networks with the corresponding phylogenetic analyses rather than showing the first ones in separate figures.

>> Thanks a lot for the positive comments and suggestions. We have corrected the text where we have misused of the terms parasite and disease as suggested, and intergrated the haplotype networks with corresponding phylogenetic analyses.
--------------------

PLOS authors have the option to publish the peer review history of their article (<a href="<https://journals.plos.org/plosntds/s/editorial-and-peer-review-process#loc-peer-review-history>" target="_blank">what does this mean?</a>). If published, this will include your full peer review and any attached files.<br><br>

If you choose “no”, your identity will remain anonymous but your review may still be made public.<br><br>

<b>Do you want your identity to be public for this peer review?</b> For information about this choice, including consent withdrawal, please see our <a href="<https://www.plos.org/privacy-policy>" target="_blank">Privacy Policy</a>.

Reviewer #1: No

Reviewer #2: No

Reviewer #3: No


Figure Files:
While revising your submission, please upload your figure files to the Preflight Analysis and Conversion Engine (PACE) digital diagnostic tool, [https://pacev2.apexcovantage.com](https://pacev2.apexcovantage.com/). PACE helps ensure that figures meet PLOS requirements. To use PACE, you must first register as a user. Then, login and navigate to the UPLOAD tab, where you will find detailed instructions on how to use the tool. If you encounter any issues or have any questions when using PACE, please email us at [figures@plos.org](mailto:figures@plos.org).
 
Data Requirements:
Please note that, as a condition of publication, PLOS' data policy requires that you make available all data used to draw the conclusions outlined in your manuscript. Data must be deposited in an appropriate repository, included within the body of the manuscript, or uploaded as supporting information. This includes all numerical values that were used to generate graphs, histograms etc.. For an example see here: <http://www.plosbiology.org/article/info%3Adoi%2F10.1371%2Fjournal.pbio.1001908#s5>.
 
Reproducibility:
To enhance the reproducibility of your results, we recommend that you deposit your laboratory protocols in [protocols.io](http://protocols.io/), where a protocol can be assigned its own identifier (DOI) such that it can be cited independently in the future. Additionally, PLOS ONE offers an option to publish peer-reviewed clinical study protocols. Read more information on sharing protocols at <https://plos.org/protocols?utm_medium=editorial-email&utm_source=authorletters&utm_campaign=protocols>

__________________________________________________
In compliance with data protection regulations, you may request that we remove your personal registration details at any time.  (Use the following URL: <https://www.editorialmanager.com/pntd/login.asp?a=r>). Please contact the publication office if you have any questions.
